# Prognostic consequences of borderline dysnatremia: pay attention to minimal serum sodium change

**DOI:** 10.1186/cc11937

**Published:** 2013-01-21

**Authors:** Michael Darmon, Eric Diconne, Bertrand Souweine, Stéphane Ruckly, Christophe Adrie, Elie Azoulay, Christophe Clec'h, Maïté Garrouste-Orgeas, Carole Schwebel, Dany Goldgran-Toledano, Hatem Khallel, Anne-Sylvie Dumenil, Samir Jamali, Christine Cheval, Bernard Allaouchiche, Fabrice Zeni, Jean-François Timsit

**Affiliations:** 1 Medical ICU, Saint-Etienne University Hospital, Avenue Albert Raymond, 42270, Saint-Priest-en-Jarez, France; Jacques Lisfranc Faculty of Medicine, Jean Monnet University, 15 Rue Ambroise Paré, 42023, Saint-Etienne, France; 2 Medical ICU, Gabriel Montpied University Hospital, 58 Rue Montalembert, 63003, Clermont-Ferrand Cedex 1, France; 3 University of Grenoble 1 (Joseph Fourier) Integrated Research Center U 823 - Albert Bonniot Institute, Grenoble University Hospital, Rond Point de la Chantourne, 38706, La Tronche, Grenoble, France; 4 Polyvalent ICU, Grenoble University Hospital, Pavillon Dauphine, BP217, 38043, Grenoble Cedex 9, France; 5 Department of Physiology, Cochin University Hospital, APHP, 27 Rue du Faubourg St Jacques, 75014, Paris, France; 6 Medical ICU, St Louis University Hospital, 1 Avenue Claude Vellefaux, 75010, Paris, France; 7 Medical-Surgical ICU, Avicenne University Hospital, 125 Rue de Stalingrad, 93000, Bobigny, France; Paris-13 University, 93000, Bobigny, France; 8 Polyvalent ICU, Groupe Hospitalier St Joseph, 145 Rue Raymond Losserand, 75014, Paris, France; 9 Polyvalent ICU, Gonesse General Hospital, 25 Rue Bernard Fevrier, 95500, Gonesse, France; 10 Intensive Care Unit, Centre Hospitalier Andrée Rosemon, Avenue des Flamboyants, 97306, Cayenne, France; 11 Surgical ICU, Antoine Béclère University Hospital, 157 Rue de la Porte de Trivaux, 92141, Clamart Cedex, France; 12 Polyvalent ICU, Centre Hospitalier Sud Essonne Dourdan-Etampes-Siège, 26 Avenue Charles de Gaulle, 91150, Etampes, France; 13 Medical-Surgical ICU, Hyeres Hospital, Rue du Maréchal Juin, 83407, Hyeres, France; 14 Surgical ICU, Edouard Herriot University Hospital, Hospices Civiles de Lyon, 5 Place Arsonval, 69437, Lyon, France

## Abstract

**Introduction:**

To assess the prevalence of dysnatremia, including borderline changes in serum sodium concentration, and to estimate the impact of these dysnatremia on mortality after adjustment for confounders.

**Methods:**

Observational study on a prospective database fed by 13 intensive care units (ICUs). Unselected patients with ICU stay longer than 48 h were enrolled over a 14-year period were included in this study. Mild to severe hyponatremia were defined as serum sodium concentration < 135, < 130, and < 125 mmol/L respectively. Mild to severe hypernatremia were defined as serum sodium concentration > 145, > 150, and > 155 mmol/L respectively. Borderline hyponatremia and hypernatremia were defined as serum sodium concentration between 135 and 137 mmol/L or 143 and 145 respectively.

**Results:**

A total of 11,125 patients were included in this study. Among these patients, 3,047 (27.4%) had mild to severe hyponatremia at ICU admission, 2,258 (20.3%) had borderline hyponatremia at ICU admission, 1,078 (9.7%) had borderline hypernatremia and 877 (7.9%) had mild to severe hypernatremia. After adjustment for confounder, both moderate and severe hyponatremia (subdistribution hazard ratio (sHR) 1.82, 95% CI 1.002 to 1.395 and 1.27, 95% CI 1.01 to 1.60 respectively) were associated with day-30 mortality. Similarly, mild, moderate and severe hypernatremia (sHR 1.34, 95% CI 1.14 to 1.57; 1.51, 95% CI 1.15 to 1.99; and 2.64, 95% CI 2.00 to 3.81 respectively) were independently associated with day-30 mortality.

**Conclusions:**

One-third of critically ill patients had a mild to moderate dysnatremia at ICU admission. Dysnatremia, including mild changes in serum sodium concentration, is an independent risk factor for hospital mortality and should not be neglected.

## Introduction

Dysnatremia is a common finding at ICU admission [1-3]. Abnormal serum sodium concentrations are known to adversely affect physiologic function and an increasing body of evidence suggests that dysnatremia may be associated with adverse outcome [1-4]. Critically ill patients are particularly exposed to dysnatremia due to the nature of the disease leading to ICU admission and to lack of free access to water [2,4,5]. Up to one-third of critically ill patients have a dysnatremia at ICU admission [2]. In addition, another one-third of critically ill patients will develop an ICU-acquired dysnatremia during ICU stay [4,6]. Prevalence of dysnatremia at ICU admission, however, varies greatly according to the chosen definition [1,2,7,8].

Doubts exist regarding prognostic impact of borderline changes in serum sodium concentration. Serum sodium concentration is closely regulated and physiological serum sodium concentration ranges between 138 and 142 mmol per liter [9] whereas abnormal serum concentration are usually defined as serum sodium lower than 135 or higher than 145 mmol per liter [2]. Most of the studies performed to date chose to focus on severe dysnatremia [1,5,7,10]. Recently, Funk and colleagues demonstrated influence of dysnatremia at ICU admission to be independently associated with outcome [2]. In this study, mild dysnatremia was independently associated with a poor outcome [2]. In addition, the association between serum sodium concentration and prognosis followed a U shape [2]. According to this observation, we hypothesized that borderline and mild changes in serum sodium concentration, lower than changes that usually alert physicians, should be taken into account.

The objective of this study was to assess the prevalence of dysnatremia, including borderline changes in serum sodium concentration in a large multicenter cohort of patients and to confirm the association of mild or moderate abnormal serum sodium concentration with mortality.

## Materials and methods

### Study design and data source

We conducted a retrospective study on a prospective multicenter database (OutcomeRea™) to assess the epidemiological characteristics and prognostic impact of dysnatremia. This study was approved by our institutional review board (CECIC Clermont-Ferrand - IRB number 5891; Ref: 2007-16) according to the French regulation on non-interventional studies which waived the need for signed informed consent for patients included in this database. Patients and patients' next of kin were, however, consulted for their willingness to decline participation to this database, and none refused to participate. The database, fed by 13 French ICUs, collects prospective data on daily disease severity, iatrogenic events, and nosocomial infections. Each year, each ICU includes a random sample of at least 50 patients who have ICU stays longer than 24 h. Each ICU could choose to obtain the random sample by taking either consecutive admissions to selected ICU beds throughout the year or consecutive admissions to all ICU beds for 1 month.

### Study population and definitions

We included consecutive patients who met the following criteria: age older than 18 years, entry in the database between January 1997 and April 2011. Patients without serum sodium measurement at ICU admission or with ICU stay of less than 48 h were secondarily excluded from the study.

Serum sodium concentration is reported at ICU admission.

We defined normal serum sodium concentration as a serum sodium level between 138 and 142 mmol/L [9].

Borderline dysnatremia were defined as serum sodium concentration between 135 and 137 mmol/L and between 143 and 145 mmol/L for borderline hyponatremia and for borderline hypernatremia respectively.

Hyponatremia were defined as serum sodium concentration < 135 and ≥ 130, < 130 and ≥ 125 or < 125 for mild, moderate and severe hyponatremia respectively [6].

Hypernatremia were defined as serum sodium concentration > 145 and ≤ 150, > 150 and ≤ 155 or > 155 for mild, moderate and severe hypernatremia respectively [6].

### Data collection

Data were collected daily by senior physicians and/or specifically trained study monitors in the participating ICUs. For each patient, the investigators entered the data into a computer case-report form using data-capture software (RHEA; OutcomeRea™, France) and imported all records into the OutcomeRea™ database. All codes and definitions were established prior to study initiation. The data quality checking procedure has been already described elsewhere [11]. The following information was recorded: age and sex, admission category (medical, scheduled surgery, or unscheduled surgery), origin (home, ward, or emergency department). Severity of illness was evaluated on the first ICU day using the Simplified Acute Physiology Score (SAPS) II, Sequential Organ Failure Assessment (SOFA) score and the Logistic Organ Dysfunction (LOD) score [12-14]. Knaus scale definitions were used to record preexisting chronic organ failures including respiratory, cardiac, hepatic, renal, and immune system failure [15].

### Quality of the database

For most of the study variables, the data-capture software immediately ran an automatic check for internal consistency, generating queries that were sent to the ICUs for resolution before incorporation of the new data into the database. In each participating ICU, data quality was checked by having a senior physician from another participating ICU review a 2% random sample of the study data every other year. A 1-day data-capture training course held once a year was open to all the OutcomeRea™ investigators and study monitors. All qualitative variables used in the analyses had κ coefficients > 0.8 and all variables had inter-rater coefficients in the 0.67 to 1 range, indicating good to excellent reproducibility.

### Statistical analysis

Values of categorical variables are reported as numbers (%) and values of continuous variables as medians (interquartile range, IQR). The chi-square test was used for categorical data and the Wilcoxon test for continuous data.

Potential risk factors for dysnatremia were entered in a Fine and Gray extension of a Cox model. Then, we used the Fine and Gray subdistribution hazard regression model [16], with day-30 mortality as the variable of interest. Discharge alive from the ICU was handled as a competing event. Subdistribution hazard ratios (sHR) and 95% confidence intervals (95% CI) were calculated.

*P *values < 0.05 were considered significant. Analyses were performed using SAS 9.1 software (SAS Institute; Cary, NC, USA).

## Results

### Study population

**Figure 1:**
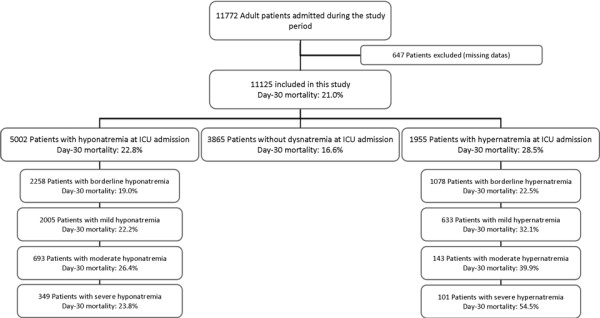
**Flow chart of patients admitted during the study period**.

**Figure 2:**
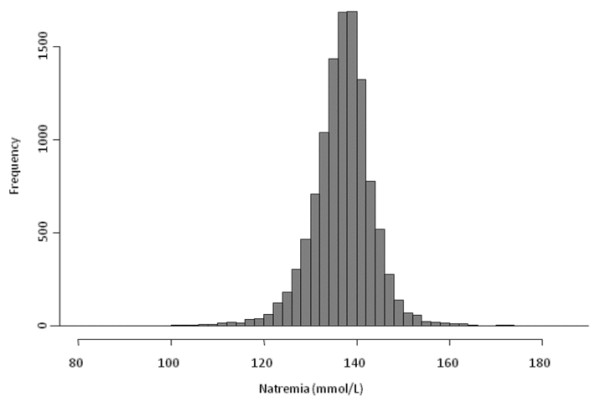
**Natremia distribution at ICU admission**.

Of the 11,772 patients with ICU stays longer than 48 h who were entered into the database during the study period, 647 (5.5%) were excluded because of missing data (Figure 1). A total of 11,125 patients were included in this study. Some 3,047 (27.38%) had mild to severe hyponatremia at ICU admission, 2,258 (20.30%) had borderline hyponatremia at ICU admission, 1,078 (9.69%) had borderline hypernatremia and 877 (7.88%) had mild to severe hypernatremia (Figure 1). Among patients with mild to severe hyponatremia, 2,005 (18.02% of overall population) had mild hyponatremia, 693 (6.23% of overall population) had moderate hyponatremia, and 349 (3.14% of overall population) had severe hyponatremia. Among patients with mild to severe hypernatremia, 633 (5.69% of overall population) had mild hypernatremia, 143 (1.29% of overall population) had moderate hypernatremia, and 101 (0.91%) had severe hypernatremia (Figure 1 and Additional file 1, Figure S1). Serum sodium value at ICU admission is reported in Figure 2.

### Characteristics of patients

Characteristics of patients at ICU admission are reported in Table 1. Overall, patients with dysnatremia were older, less frequently of male gender, had a higher body weight at ICU admission, a greater disease severity as assessed by SAPS II, SOFA score, organ failure at ICU admission or need for supportive therapy. In addition, patients with dysnatremia had more frequently underlying chronic illnesses.

**Table 1:** Characteristics of patients.

	Severe hypoNa Na < 125	Moderate hypoNa 125 ≤ Na < 130	Mild hypoNa 130 ≤ Na < 135	Borderline hypoNa 135 ≤ Na < 138	No dysnatremia 138-142	Borderline hyperNa 142 > Na ≥ 145	Mild hyperNa 145 > Na ≥ 150	Moderate hyperNa 150 > Na ≥ 155	Severe hyperNa Na > 155	*P *value*
	*N *= 349	*N *= 693	*N *= 2005	*N *= 2258	*N *= 3865	*N *= 1078	*N *= 633	*N *= 143	*N *= 101	
**Patient characteristics**										
Male gender	197 (56.4%)	392 (56.6%)	1211 (60.4%)	1381 (61.2%)	2391 (61.9%)	649 (60.2%)	380 (60%)	81 (56.6%)	60 (59.4%)	0.1846
Age (yrs)	62 (51-75)	63 (49.5-75.5)	63 (51-76)	62 (49-75)	63 (49-76)	62 (48-75)	65 (53-76)	67 (46-77)	64 (57-77)	0.0358
Weight (kg)	67 (56-80)	69 (58-81)	68 (58.4-80)	71 (60-83)	70.3 (60-83)	70 (60-82)	70 (59.7-81)	65 (59.7-80)	69 (55-80)	< .0001
SOFA score [13]	6 (3-8)	6 (4-9)	5 (3-8)	5 (3-7)	5 (2-7)	5 (3-9)	7 (4-10)	8 (5-11)	8 (6-12)	< .0001
SAPS II score [14]	56 (45-66)	57 (47-66)	55 (44-65)	51 (41-61)	50 (39-60)	53 (43-65)	57 (47-69)	63 (52-73)	66 (54-78)	< .0001
**Underlying condition**										
Chronic kidney disease	20 (5.7%)	50 (7.2%)	142 (7.1%)	121 (5.4%)	225 (5.8%)	61 (5.7%)	30 (4.7%)	7 (4.9%)	4 (4%)	0.2031
Immunocompromised	55 (15.8%)	143 (20.6%)	366 (18.3%)	343 (15.2%)	458 (11.8%)	128 (11.9%)	93 (14.7%)	12 (8.4%)	12 (11.9%)	< .0001
**Main symptom at admission**										
Acute respiratory failure	65 (18.6%)	189 (27.3%)	529 (26.4%)	580 (25.7%)	893 (23.1%)	244 (22.6%)	151 (23.9%)	21 (14.7%)	20 (19.8%)	0.0003
Coma	68 (19.5%)	74 (10.7%)	216 (10.8%)	296 (13.1%)	661 (17.1%)	236 (21.9%)	138 (21.8%)	51 (35.7%)	28 (27.7%)	< .0001
Septic shock	33 (9.5%)	98 (14.1%)	294 (14.7%)	225 (10%)	343 (8.9%)	107 (9.9%)	76 (12%)	18 (12.6%)	9 (8.9%)	< .0001
Shock (other)	42 (12%)	86 (12.4%)	226 (11.3%)	218 (9.7%)	327 (8.5%)	122 (11.3%)	84 (13.3%)	16 (11.2%)	16 (15.8%)	< .0001
Acute renal failure	47 (13.5%)	78 (11.3%)	128 (6.4%)	101 (4.5%)	108 (2.8%)	39 (3.6%)	25 (3.9%)	7 (4.9%)	7 (6.9%)	< .0001
Trauma	0	1 (0.1%)	13 (0.6%)	19 (0.8%)	66 (1.7%)	11 (1%)	6 (0.9%)	5 (3.5%)	1 (1%)	< .0001
**Treatments at ICU admission**										
Antibiotics	190 (54.4%)	446 (64.4%)	1278 (63.7%)	1236 (54.7%)	1903 (49.2%)	567 (52.6%)	387 (61.1%)	90 (62.9%)	75 (74.3%)	< .0001
Central venous catheter	125 (35.8%)	312 (45%)	824 (41.1%)	867 (38.4%)	1446 (37.4%)	459 (42.6%)	342 (54%)	79 (55.2%)	60 (59.4%)	< .0001
Vasoactive drugs	107 (30.7%)	259 (37.4%)	671 (33.5%)	650 (28.8%)	1041 (26.9%)	350 (32.5%)	277 (43.8%)	65 (45.5%)	49 (48.5%)	< .0001
Mechanical ventilation	124 (35.5%)	253 (36.5%)	840 (41.9%)	1026 (45.4%)	1846 (47.8%)	605 (56.1%)	363 (57.3%)	93 (65%)	62 (61.4%)	< .0001
Renal replacement therapy	53 (15.2%)	113 (16.3%)	212 (10.6%)	217 (9.6%)	307 (7.9%)	96 (8.9%)	83 (13.1%)	11 (7.7%)	19 (18.8%)	< .0001
**Outcome**										
DFLST	16 (4.6%)	49 (7.1%)	108 (5.4%)	93 (4.1%)	171 (4.4%)	78 (7.2%)	51 (8.1%)	11 (7.7%)	7 (6.9%)	< .0001
ICU mortality	66 (18.9%)	160 (23.1%)	381 (19%)	360 (15.9%)	551 (14.3%)	220 (20.4%)	176 (27.8%)	48 (33.6%)	49 (48.5%)	< .0001
Day-30 mortality	83 (23.8%)	183 (26.4%)	446 (22.2%)	430 (19.0%)	642 (16.6%)	242 (22.5%)	203 (32.1%)	57 (39.9%)	55 (54.5%)	< 0.001
Hospital mortality	89 (25.5%)	201 (29.0%)	483 (24.1%)	458 (20.3%)	693 (17.9%)	264 (24.5%)	215 (34.0%)	58 (40.6%)	57 (56.4%)	< .0001

*Comparison across different subclasses of serum sodium concentration at ICU admission. The data are reported as medians (IQR). SOFA, Sequential Organ Failure Assessment, which can range from 0 to 24; SAPS II, Simplified Acute Physiology Score version II, which can range from 0 to 155; DFLST, decision to forgo life-sustaining treatments.

### Outcome of patients with hypernatremia at ICU admission

**Figure 3:**
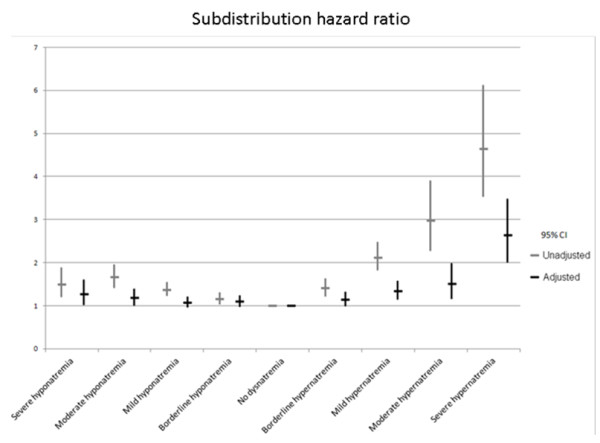
**Relationship between hospital admission serum sodium concentrations and day-30 mortality**. Subdistribution hazard ratio (sHR) and 95% confidence interval (95% CI) are represented before (light grey) and after adjustment for confounders (dark).

Before adjustment, mortality at day 30 was 21.0% in the overall population of patients. Crude hospital mortality was increased in patients with borderline to severe hypernatremia and in patients with borderline to severe hyponatremia (Figure 1 and 3). Day-30 mortality was of 16.6% in patients without dysnatremia at ICU admission, 19.0% in patients with borderline hyponatremia, 22.2% in patients with mild hyponatremia, 26.4% in patients with moderate hyponatremia and 23.8% in patients with severe hyponatremia (Table 1 and Figure 3). Day-30 mortality was 22.5% in patients with borderline hypernatremia, 32.1% in patients with mild hypernatremia, 39.9% in patients with moderate hypernatremia and 54.5% in patients with severe hypernatremia. Cumulated incidence of mortality according to serum sodium concentration at ICU admission is reported in Additional file 1, Figure S2a (hyponatremia) and Additional file 1, Figure S2b (hypernatremia). Relationship between serum sodium concentration and day-30 mortality is reported in Additional file 1, Figure S3.

When entered into a Fine and Gray model (Table 2), moderate to severe hyponatremia (sHR 1.82, 95% CI 1.002 to 1.395 and sHR 1.27, 95% CI 1.01 to 1.60 for moderate and severe hyponatremia respectively) and mild to severe hypernatremia (sHR 1.34, 95% CI 1.14 to 1.57; sHR 1.51, 95% CI 1.15 to 1.99; and sHR 2.64, 95% CI 2.00 to 3.81 for mild, moderate and severe hypernatremia respectively) remained independently associated with day-30 mortality. Borderline hyponatremia (sHR 1.09, 95% CI 0.97 to 1.23; *P *= 0.15) and borderline hypernatremia (sHR 1.14, 95% CI 0.99 to 1.33; *P *= 0.08) were not associated with day-30 mortality after adjustment for confounders. Figure 3 reports adjusted association between different class of dysnatremia and day-30 mortality before and after adjustment.

**Table 2:** Factors independently associated with day-30 mortality.

	sHR	95% CI	*P *value
**Natremia**			
**Severe hyponatremia**	**1.27**	**(1.01-1.60)**	**0.040**
**Moderate hyponatremia**	**1.18**	**(1.002-1.40)**	**0.047**
Mild hyponatremia	1.08	(0.95-1.22)	0.23
Borderline hyponatremia	1.09	(0.97-1.24)	0.15
*Normal natremia*	*1*	*(Ref)*	-
Borderline hypernatremia	1.09	(0.97-1.24)	0.15
**Mild hypernatremia**	**1.34**	**(1.14-1.57)**	**0.0003**
**Moderate hypernatremia**	**1.51**	**(1.15-1.99)**	**0.003**
**Severe hypernatremia**	**2.64**	**(2.00-3.48)**	**< 0.0001**
DFLST*	**3.24**	**(2.90-3.62)**	**< 0.0001**
**Intoxication as reason for ICU admission**	**0.21**	**(0.13-0.34)**	**< 0.0001**
**Chronic cardiac dysfunction**	**1.26**	**(1.13-1.41)**	**< 0.0001**
**Immunocompromised patient**	**1.30**	**(1.17-1.45)**	**< 0.0001**
**Age > 64 yrs**	**1.56**	**(1.43-1.71)**	**< 0.0001**
SOFA score (per point)*	**1.21**	**(1.20-1.23)**	**< 0.0001**

(Subdistribution hazard ratios (sHR) and 95% confidence intervals (95% CI)). *DFLST, decision to forgo life-sustaining treatments; SOFA, Sequential Organ Failure Assessment, which can range from 0 to 24.

## Discussion

This large multicenter cohort study, focusing specifically on dysnatremia at ICU admission, demonstrates that this electrolyte disorder is common and is an independent risk factor for ICU mortality. This study confirms that mild hypernatremia and moderate hyponatremia (that is serum sodium concentration < 130 mmol/L or > 145 mmol/L respectively) are independently associated with poor outcome.

In this study, one-third of critically ill patients had a moderate to severe dysnatremia at ICU. Mild to severe hypernatremia and hyponatremia were present in respectively 7.8% and 27.4% of the admitted patients. Hyponatremia is the most common electrolyte disorder in hospitalized patients. Up to 40% of the overall population of hospitalized patients has a hyponatremia at admission [17]. Presence of severe hyponatremia on hospital admission has been demonstrated to be independently associated with an increased risk for ICU admission and poor prognosis [18]. Hyponatremia may be due to chronic organ dysfunctions (that is heart failure or liver dysfunction) but also to diuretic use, syndrome of inappropriate antidiuretic hormone (ADH) secretion, adrenal insufficiency, and cerebral or renal salt wasting syndromes [9,19]. Each of these conditions is frequently encountered in critically ill patients and may explain the high prevalence of hyponatremia at ICU admission. Similarly, although less frequent than hyponatremia, several studies demonstrated hypernatremia to be common at ICU admission [4-7]. Since thirst and free access to water are the most important mechanisms that prevent hypernatremia, critically ill patients and older patients are at high risk for this disorder [4,19]. Only 2.5% patients have been found to develop moderate to severe hypernatremia in the general in-hospital population of patients. Higher prevalence has been reported in geriatric or critically ill patients [20-23].

In keeping with previous findings, our study demonstrates a close association between dysnatremia and hospital mortality [1-3,8,13,24]. After adjustment for patients' severity, increasing degree of dysnatremia was associated with increasing prognostic impact. Interestingly, our study demonstrates that mild to severe hypernatremia and moderate to severe hyponatremia are independently associated with outcome. Earlier studies reported an association between dysnatremia and hospital mortality [1-3,10]. However, most of these studies evaluated the more severe patients [1-3,10]. Recent studies suggested mild to moderate changes in serum sodium concentration to be associated with prognosis [2,24]. In our study, after adjustment for comorbidities, case mix, or patients' severity, even mild hypernatremia and moderate hyponatremia were independently associated with poor outcome. Hypernatremia has multiple adverse effects that may explain this association. Hypernatremia has been shown to be associated wit peripheral insulin resistance, impaired hepatic lactate clearance, decreased left ventricular contractility or various neuromuscular manifestations ranging from muscle weakness to delayed weaning from mechanical ventilation [25-28]. Similarly, hyponatremia may be associated with dismal neurological manifestations although occurring in profound hyponatremia [9,19]. Our study, however, was not designed to evaluate these consequences and no causality relationship can be drawn from this association. Last, our study was unable to demonstrate an association between outcome and borderline changes in natremia or mild hyponatremia. Nevertheless, progressive association between serum sodium changes and hospital mortality suggest that we should pay attention to mild abnormal serum sodium concentration.

Our study has several limitations. First, our study design did not allow us to evaluate the causes of dysnatremia. No data regarding fluid balance or diuretic therapy before ICU admission were available. In addition, as previously stated, our study was not designed to evaluate factors leading to this association. Although a causal role for dysnatremia in death is biologically plausible, we cannot determine from our data whether the association between dysnatremia and mortality reflected a direct effect of dysnatremia, a surrogate marker for underlying comorbidities or reason for ICU admission, or both. Further studies evaluating the influence of dysnatremia correction on prognosis or the influence of therapeutic intervention in patients with mild to moderate dysnatremia may help in answering this clinical question.

## Conclusions

Our results confirm the high prevalence of dysnatremia at ICU admission and demonstrate that even mild to moderate abnormal serum sodium concentrations are independent risk factors for ICU mortality. Although we must acknowledge that dysnatremia may be a surrogate for patients' severity, treatment or case mix, we believe that mild abnormal serum sodium concentration should not be neglected. Further studies are needed to understand factors leading to this prognostic association and the potential benefit from therapeutic strategies aiming at this metabolic disturbance.

## Key messages

• Dysnatremia is common at ICU admission. Mild to severe hypernatremia and hyponatremia were present in respectively 7.8% and 27.4% of the critically ill patients.

• Dysnatremia is independently associated with ICU mortality. In our study, mild hypernatremia (that is serum sodium concentration > 145 mmol/L) and moderate hyponatremia (that is serum sodium concentration < 130 mmol/L) are independently associated with poor outcome (respective sHR of 1.34 (95% CI 1.14 to 1.57) and 1.18 (95% CI 1.002 to 1.40)).

• Although a causal role for dysnatremia in death is biologically plausible, we cannot determine from our data whether the association between dysnatremia and mortality reflected a direct effect of dysnatremia, a surrogate marker for underlying comorbidities or reason for ICU admission, or both.

## Abbreviations

95% CI: 95% confidence interval; ICU: intensive care unit; SAPS II: Simplified Acute Physiology Score version II; sHR: subdistribution hazard ratio; SOFA: Sequential Organ Failure Assessment.

## Competing interests

The authors have no competing interests to declare.

## Authors' contributions

Prof Darmon and Prof Timsit had full access to all of the data in the study and take responsibility for the integrity of the data and the accuracy of the data analysis. MD participated in the study concept and design, the acquisition of date, the interpretation of the data, and the drafting of the manuscript. ED participated in the study design, the acquisition of the data and critical revisions of the manuscript. BS participated in the study design, the acquisition of the data and critical revisions of the manuscript. SR participated in the statistical analysis and critical revision of the manuscript. CA, EA, CC, MGO, CS, DGT, HK, ASD, SJ, CCh and BA participated in the acquisition of the data, interpretation of the results and critical revisions of the manuscript. FZ participated in the study design, the acquisition of the data and critical revisions of the manuscript. JFT participated in the study design, acquisition of date, statistical analysis, interpretation of the data, and drafting of the manuscript. The final version of the manuscript was read and approved by all of the authors.

## Supplementary Material

Additional file 1 **Additional figures and members of the Outcomerea study group**. **Figure S1: **Frequency of dysnatremia for each of the evaluated subgroups. **Figure S2**: Cumulative incidence (Y) for mortality according to serum sodium concentration at ICU admission in patients with hyponatremia **(2a) **and hypernatremia **(2b)**. **Figure S3**. Relationship between hospital admission serum sodium concentrations and day-30 mortality. **Appendix A**: Members of the Outcomerea study group. Click here for file
