# Supplementary material for: Prognostic consequences of borderline dysnatremia: pay attention to minimal serum sodium change
Source: Crit Care. 2013 Jan 21;17(1):R12. doi: 10.1186/cc11937 (PMC4056804; doi:10.1186/cc11937)
Supplement: Additional file 1 — Additional figures and members of the Outcomerea study group. Figure S1: Frequency of dysnatremia for each of the evaluated subgroups. Figure S2: Cumulative incidence (Y) for mortality according to serum sodium concentration at ICU admission in patients with hyponatremia (2a) and hypernatremia (2b). Figure S3. Relationship between hospital admission serum sodium concentrations and day-30 mortality. Appendix A: Members of the Outcomerea study group. [file cc11937-S1.DOC]

**Prognostic consequences of borderline dysnatremia: Pay attention to minimal serum sodium change**

Michael DARMON et al.

**Additional File 1.**

**Additional file 1, Figure S1. Frequency of dysnatremia for each of the evaluated subgroups**

**Additional file 1, Figure S2. Cumulative incidence (Y) for mortality according to serum sodium concentration at ICU admission in patients with hyponatremia (S2a) and hypernatremia (S2b)**

**S2a-**

**S2b-**

**Additional file 1, Figure S3. Relationship between hospital admission serum sodium concentrations and Day-30 mortality.** Grey lines represent the 95% confidence interval. Influence of natremia on day-30 mortality is reported as subdistribution hazard ratio.

**APPENDIX: Member of the Outcomerea Study Group**

**Scientific committee**

Jean-François Timsit (Hôpital Albert Michallon and Grenoble 1 university U823, Grenoble, France), Pierre Moine (Surgical ICU, Denver, Colo), Elie Azoulay (Medical ICU, Hôpital Saint Louis, Paris, France), Yves Cohen (ICU, Hôpital Avicenne, Bobigny, France), Michael Darmon (Medical-Surgical ICU, Hopital Nord, Saint-Etienne University Hospital, France), Maïté Garrouste-Orgeas (ICU Hôpital Saint- Joseph, Paris, France), Lilia Soufir (ICU, Hôpital Saint-Joseph, Paris, France), Jean-Ralph Zahar (Department of Microbiology, Hôpital Necker, Paris, France), Christophe Adrie (Department of Physiology,Hôpital Cochin,

France), Adel Benali (Microbiology and Infectious Diseases, Hôpital Saint-Joseph, Paris, France), Christophe Clec'h (ICU, Hôpital Avicenne, Bobigny, France), and Jean Carlet (ICU, Hôpital Saint-Joseph, Paris, France).

**Biostatistical and informatics expertise**

Jean-Francois Timsit (Epidemiology of Cancer and Severe Illnesses, Grenoble 1 university U823, Grenoble, France), Sylvie Chevret (Medical Computer Sciences and Biostatistics Department, Hôpital Saint-Louis, Paris, France), Corinne Alberti (Medical Computer Sciences and Biostatistics Department, Robert Debré, Paris, France), Adrien Français (Epidemiology of Cancer and Severe Illnesses, Grenoble 1 university U823, Grenoble, France), Aurélien Vésin (Epidemiology of Cancer and Severe Illnesses, Grenoble 1 university U823, Grenoble, France), Sylvain Anselme (Epidemiology of Cancer and Severe Illnesses, Grenoble 1 university, Grenoble, France), Muriel Tafflet (Outcomerea, France), Frederik Lecorre (Supelec, France), and Didier Nakache (Conservatoire National des Arts et Métiers, Paris, France).

**Investigators of the Outcomerea database**

Christophe Adrie (Department of Physiology, Hôpital Cochin, France),Bernard Allaouchiche (Surgical ICU, Hôpital Edouard Herriot, Lyon), Claire Ara-Somohano (University hospital A Michallon, Grenoble,

France ), Agnès Bonadona (University hospital A Michallon, Grenoble, France ), Caroline Bornstain (ICU, Hôpital de Montfermeil, France), Alexandre Boyer (ICU, Hôpital Pellegrin, Bordeaux, France), Antoine Caubel (ICU, Hôpital Saint-Joseph, Paris, France), Anh-Tuan Dinh-Xuan (Department of Physiology, Hôpital Cochin, France), Christine Cheval (SICU, Hôpital Saint-Joseph, Paris, France), Jean-Pierre Colin (ICU, Hôpital de Dourdan, Dourdan, France), Michael Darmon (Medical-Surgical ICU, Hopital Nord, Saint-Etienne University Hospital, France), Anne-Sylvie Dumenil (Hôpital Antoine Béclère, Clamart France), Adrien Descorps-Declere (Hôpital Antoine Béclère, Clamart France), Jean-Philippe Fosse (ICU, Hôpital Avicenne, Bobigny, France), Rebecca Hamidfar-Roy (University hospital A Michallon, Grenoble, France ), Samir Jamali (ICU, Hôpital de Dourdan, Dourdan, France), Christian Laplace (ICU, Hôpital Kremlin-Bicêtre, Bicêtre, France), Thierry Lazard (ICU, Hôpital de la Croix Saint-Simon, Paris, France), Eric Le Miere (ICU, Hôpital Louis Mourier, Colombes, France), Laurent Montesino (ICU, Hôpital Bichat, Paris, France), Bruno Mourvillier (ICU, Hôpital Bichat, France), Benoît Misset (ICU, Hôpital Saint-Joseph, Paris, France), Delphine Moreau (ICU, Hôpital Saint-Louis, Paris, France), Roman Mounier (ICU, Hôpital Louis Mourier, Colombes, France), Etienne

Pigné (ICU, Hôpital Louis Mourier, Colombes, France), Carole Schwebel (University hospital A Michallon, Grenoble, France), Jean-Francois Timsit (University hospital A Michallon, Grenoble, France ), Gilles Troché (Hôpital Antoine Béclère, Clamart France), Marie Thuong (Agence de Biomédicine, Saint Denis, France), Dany Golgran-Toledano (CH Gonesse, France), and François Vincent (ICU, Hôpital Avicenne, Bobigny, France).

Clinical research assistants Caroline Tournegros (Hôpital Albert Michallon), Silvia Calvino (Hôpital Albert Michallon), Loic Ferrand (Hôpital Albert Michallon), Samir Bekhouche (Hôpital Saint Louis), and Kaoutar Mellouk (Hôpital Saint-Joseph, Paris, France).
